# Soil multifunctionality and temporal variability of nutrients across vigor classes in wild apple trees (*Malus sieversii*)

**DOI:** 10.3389/fpls.2025.1711464

**Published:** 2026-01-12

**Authors:** Chen-Quan Gu, Jia-Qi Xu, Xiao-Bing Zhou, Mekhrovar Okhonniyozov, Yue-Wei Tong, Ye Tao

**Affiliations:** 1 College of Life Sciences, Anqing Normal University, Anqing, Anhui, China; 2 State Key Laboratory of Ecological Safety and Sustainable Development in Arid Lands, Xinjiang Institute of Ecology and Geography, Chinese Academy of Sciences, Urumqi, Xinjiang, China; 3 Xinjiang Field Scientific Observation Research Station of Tianshan Wild Fruit Forest Ecosystem, Xinjiang Institute of Ecology and Geography, Chinese Academy of Sciences, Yili, Xinjiang, China; 4 Xinjiang Key Laboratory of Biodiversity Conservation and Application in Arid Lands, Xinjiang Institute of Ecology and Geography, Chinese Academy of Sciences, Urumqi, Xinjiang, China; 5 Research Center for Ecology and Environment of Central Asia, Dushanbe, Tajikistan

**Keywords:** dynamic change, growth status, *M. sieversii*, meteorological factor, soil multifunctionality, temporal variability

## Abstract

*Malus sieversii*, a Tertiary relict and primary progenitor of the cultivated apple, is experiencing severe habitat degradation in China’s Tianshan Mountains. To understand how soil ecosystem functions respond to tree vigor decline, we monitored surface soils beneath the canopy of wild apple trees monthly from April to October. Trees were classified into three vigor classes based on the percentage of dead branches: Vigor Class I (<20%), Vigor Class II (40–60%), and Vigor Class III (>80%). Soil multifunctionality (SMF) and temporal variability of nutrients (TVN) were derived from seven key nutrient indicators. Soils under Vigor Class II trees exhibited the lowest SMF and highest TVN, indicating maximal functional instability during intermediate degradation. While SMF peaked and TVN reached its seasonal minimum in October, Vigor Class II showed a consistent decline in TVN over time, unlike the irregular fluctuations in Vigor Classes I and III. A significant negative SMF–TVN correlation in Vigor Classes II and III suggests a trade-off between functionality and stability. Partial least squares path modeling revealed that soil organic carbon, total nitrogen, and total phosphorus were the dominant direct driver of both SMF and TVN, with climate exerting no significant direct effects once tree vigor and soil conditions were accounted for. These results suggest that Vigor Class II represents a critical early-warning stage: soil functional capacity begins to deteriorate before visible signs of severe tree decline or mortality. Targeted ecological restoration of Vigor Class II trees is essential to prevent irreversible ecosystem degradation. Therefore, while continued protection of healthy Vigor Class I trees remains essential, conservation efforts should place greater emphasis on restoring Vigor Class II trees to disrupt degradation feedbacks before irreversible ecosystem decline occurs.

## 1 Introduction

*M. sieversii*, a Tertiary relict species endemic to the arid regions of Central Asia, is a unique wild fruit tree resource. Owing to its basal phylogenetic position, it constitutes a critical component of the global apple germplasm pool (Chu et al., 2022). As a keystone species, it helps maintain the stability of the wild fruit forest ecosystems in maintaining the stability of wild fruit forest ecosystems of the Tianshan Mountains and serves as a living fossil that provides insights into the evolutionary history of the ancient Mediterranean flora. *M. sieversii* is primarily distributed across Kazakhstan, Kyrgyzstan, and the Chinese Tianshan Mountains (Xinjiang Uygur Autonomous Region), extending into adjacent areas such as Emin and Tuoli counties. Within these regions, *M. sieversii* serves as the dominant constructive species of the Tianshan wild fruit forests. Wild apple populations harbor ancient genetic resources, with as many as 84 intraspecific types documented in Xinjiang alone. This diversity represents a vital germplasm repository in China and holds considerable scientific value for biodiversity conservation and evolutionary research (Bai et al., 1998). The fruits of wild apple exhibit primitive morphological traits, such as a large endocarp, which provide critical insights into the origin and evolutionary trajectory of domesticated apples. Recent genomic studies have confirmed that wild apples in Xinjiang, China, represent one of the primary centers of origin for cultivated apples worldwide (Velasco et al., 2010). Over the past several decades, the wild fruit forest ecosystems of the Tianshan Mountains have undergone severe degradation, driven by a combination of anthropogenic disturbances, such as farmland expansion, overgrazing, and logging, as well as biotic pressures from *Agrilus mali* and the impacts of climate change. As the dominant constructive species, *M. sieversii* populations have experienced a marked decline in both abundance and distribution range (Zhou, 2024). Within the remaining populations, ongoing mortality of mature individuals, coupled with limited seedling recruitment, has resulted in pronounced demographic decline. Consequently, natural regeneration of *M. sieversii* is severely constrained (Su et al., 2019; Zhang et al., 2022a). In the Ili region of Xinjiang, Tianshan wild fruit forests have experienced widespread dieback and large-scale mortality. The most severe cases are in Xinyuan County, where mortality rates of *M. sieversii* exceed 80% in certain areas (Yang, 2020) In response, *M. sieversii* was designated as a Class II nationally protected species under China’s wildlife conservation system in 2021.To further elucidate the mechanism underlying its decline, extensive research has been conducted on population age structure (Su et al., 2019), genetic diversity (Zhang et al., 2022a), photosynthesis-related stress responses, pest and disease impacts and their management (Mierkamili et al., 2021), pathogenic microorganisms (Li et al., 2020), and soil microbial communities (Rong et al., 2024). Nevertheless, the dynamic changes in multifunctional soil properties within wild apple vigor classes, examined from a systems ecology perspective, have not yet been reported.

Soil serves as the fundamental substrate for plant growth, and its nutrient status directly its nutrient status directly influences plant development. Soil multifunctionality (SMF) refers to the capacity of soil to simultaneously support multiple ecosystem functions, encompassing a suite of soil processes that underpin ecosystem service provision (Bünemann et al., 2018). Empirical studies have demonstrated that SMF varies in response to a range of environmental drivers, including climate (e.g., temperature and precipitation), soil properties (e.g., pH and texture), soil microbial diversity, and plant community attributes such as species richness, asynchrony, and compositional stability (Hu et al., 2021; Wang et al., 2024; Zhang et al., 2025). Moreover, SMF is inherently dynamic, exhibiting temporal shifts over the growing season or across longer time scales due to external environmental fluctuations and alterations in soil biochemical processes. For example, on the Tibetan Plateau, grassland degradation has been linked to shifts in soil multifunctionality (SMF). Wang et al. (2021) reported SMF indices in the order: non-degraded > moderately degraded > lightly degraded > severely degraded, indicating a non-monotonic response across degradation stages. In contrast, Zhou et al. (2022) observed a more consistent decline in ecosystem multifunctionality with increasing degradation severity in alpine meadows, where composite SMF scores followed the expected sequence: lightly degraded > moderately degraded > severely degraded. Despite these differences, which may arise from variations in degradation classification criteria, site-specific environmental conditions, or temporal dynamics, the overall trend underscores that severe degradation consistently impairs soil multifunctionality. Moreover, as degraded ecosystems undergo recovery, SMF typically exhibits a marked upward trajectory (Guo et al., 2021; Zhou, 2024). Therefore, these findings highlight that investigating SMF can yield critical insights into the mechanisms driving plant degradation and support the design of targeted, context-sensitive management strategies.

Complementing soil multifunctionality (SMF), the temporal stability of soil functions is a key determinant of ecosystem resilience. To quantify this stability, we defined the Temporal Variability of Nutrients (TVN) as the mean coefficient of variation (CV) across seven core soil nutrient indicators, where the CV for each indicator is calculated from spatial variation among replicate trees within each vigor class and month, serving as a proxy for temporal instability. A higher TVN thus reflects greater temporal instability in soil nutrient supply, indicating reduced functional resilience (Durán et al., 2018). Critically, increased temporal variability in ecosystem state variables is theorized to serve as an early-warning signal of impending critical transitions, as systems approaching a tipping point often exhibit heightened fluctuations due to declining stabilizing feedbacks (Scheffer et al., 2009). In systems where longitudinal monitoring is constrained, elevated TVN in moderately declined individuals relative to healthier or severely degraded counterparts may still indicate proximity to a functional threshold, even if variability does not increase over time within a given class. Empirical and methodological studies have further operationalized this theory, demonstrating that statistical indicators like rising variance can be detected in time series prior to regime shifts (Dakos et al., 2012). In this context, TVN may not merely reflect current instability but signal an elevated risk of functional collapse before mean performance (e.g., SMF) shows irreversible decline. TVN is jointly influenced by abiotic factors (e.g., climate and edaphic properties) and biotic interactions (e.g., vegetation condition and soil microbial communities) (Zheng et al., 2019; Luo et al., 2022). Under environmental stressors such as drought, TVN typically increases, signaling a loss of consistency in nutrient cycling dynamics (Berdugo et al., 2020). Despite its ecological relevance, how TVN varies across degradation gradients in long-lived species like *M. sieversii* remains unexplored. Accordingly, this study addresses two central questions:

(1) How do SMF and TVN vary dynamically across wild apple trees exhibiting differing levels of vigor? (2) What is the nature of the relationship between SMF and TVN among trees representing different degradation states?

To answer these questions, we conducted monthly surface soil sampling beneath the canopy of *M. sieversii* from April to October within the Yili Botanical Garden in Xinyuan County, Ili River Valley, across three vigor classes defined by crown dieback: relatively healthy (Class I: <20%), moderately declined (Class II: 40–60%), and severely declined (Class III: >80%).,Integrating these data with concurrent meteorological records, we characterized the seasonal dynamics of soil nutrient status, SMF, and TVN, and evaluated the relative roles of intrinsic soil properties versus climate in driving these metrics. By jointly analyzing “soil multifunctionality” and “temporal stability”, this study provides a novel framework for diagnosing ecosystem degradation in threatened wild fruit forests and offers a scientific basis for prioritizing conservation and restoration actions.

## 2 Results

### 2.1 Variation in soil nutrients across vigor classes and growth periods

**Figure 1:**
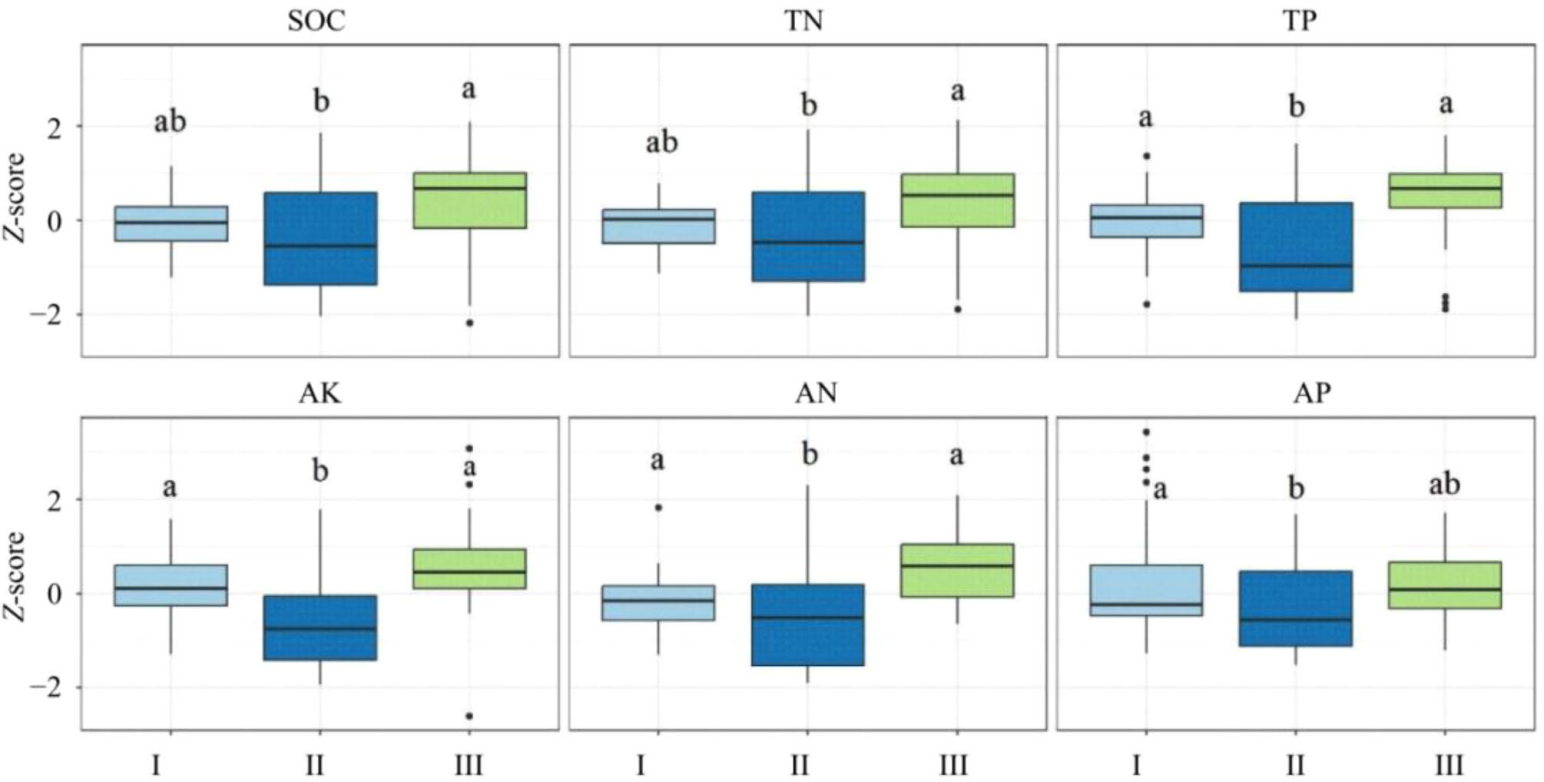
Differences in six soil nutrient indicators (standardized Z-scores) among wild apple vigor classes (I, II, and III). Only soil factors with significant differences are presented. Different lowercase letters indicate significant differences (P < 0.05). SOC, soil organic carbon; TN, total nitrogen; TP, total phosphorus; AK, available potassium; AN, available nitrogen; AP, available phosphorus.

Tree vigor class significantly influenced soil organic carbon (SOC; *P* = 0.008) and total nitrogen (TN; *P* = 0.016) (**Table 1**). Soils associated with *Malus sieversii* trees in vigor class III exhibited significantly higher SOC and TN than those in class II, with increases of 27.00% and 24.16%, respectively (**Figure 1**). No significant differences in SOC and TN were observed between class I and class II. Vigor class had a significant effect on total phosphorus (TP; *P* < 0.001), available nitrogen (AN; *P* < 0.001), available potassium (AK; *P* < 0.001), and available phosphorus (AP; *P* = 0.027). Specifically, TP concentrations in soils of trees in vigor class I and class III were significantly higher than those in class II, by 9.3% and 17.6%, respectively. Similarly, soils from *M. sieversii* trees in vigor classes I and III had significantly higher AN than those in class II, with increases of 8.25% and 37.72%, respectively. AK in class I and class III was significantly greater than in class II by 27.57% and 41.85%, respectively. For AP, soils from vigor class I showed a significant 41.33% increase compared to class II, whereas no significant difference was observed between class III and class II.

**Table 1:** Effects of growth period (month), vigor classes, and their interaction on soil nutrient parameters in wild apple vigor classes.

Soil nutrient	Month			Vigor			Month × Vigor		
	*df*	*F*	*P*	*df*	*F*	*P*	*df*	*F*	*P*
SOC	6	1.026	0.414	2	5.156	0.008	12	1.163	0.324
TN	6	0.651	0.689	2	4.366	0.016	12	0.953	0.499
TP	6	0.400	0.877	2	11.133	<0.001	12	2.144	0.022
TK	6	4.658	<0.001	2	1.598	0.209	12	0.538	0.884
AN	6	1.947	0.083	2	8.852	<0.001	12	0.147	1.000
AP	6	2.687	0.020	2	3.757	0.027	12	0.329	0.982
AK	6	13.127	<0.001	2	28.271	<0.001	12	0.480	0.921

**Figure 2:**
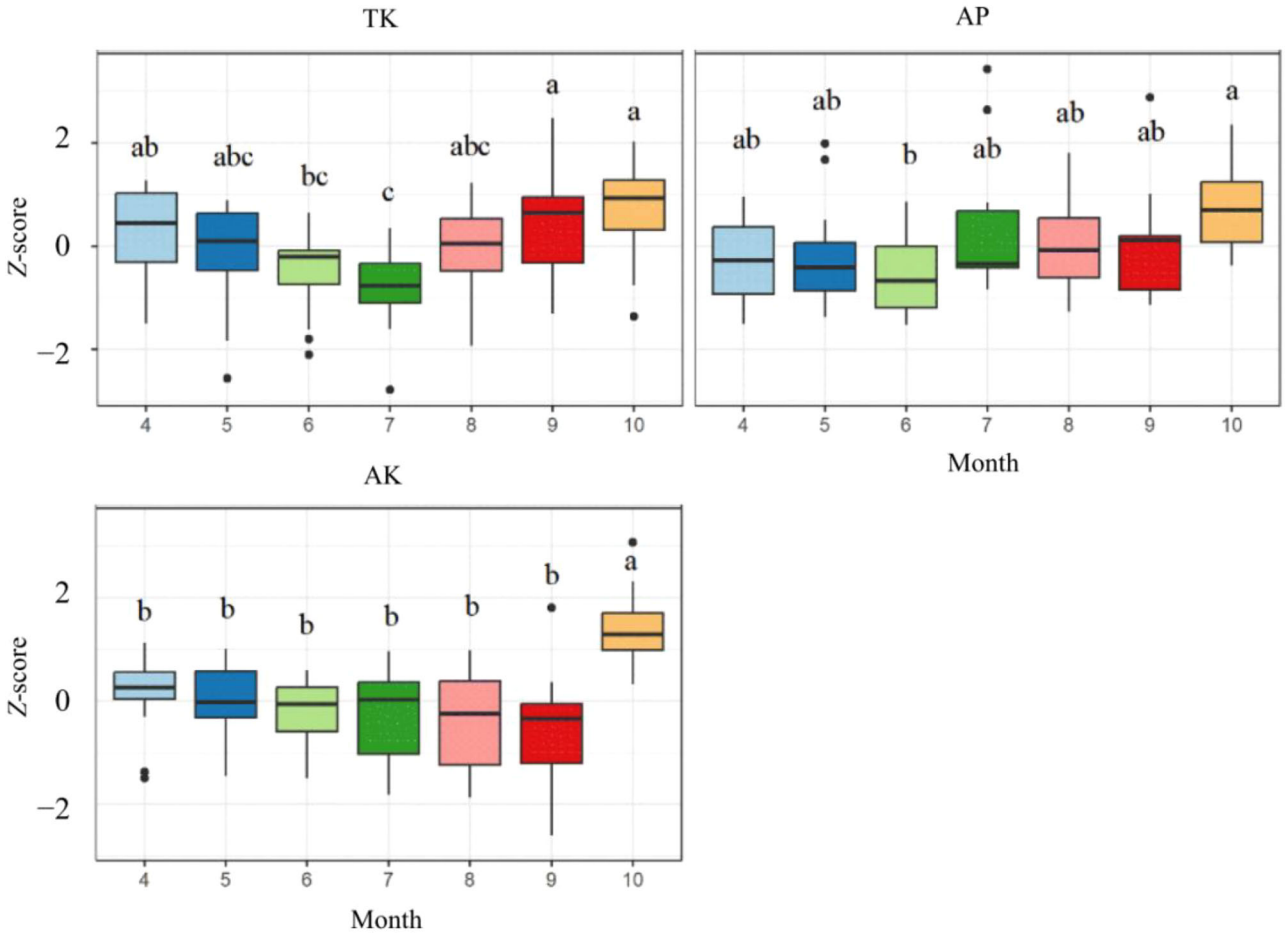
Differences in soil TK, AP and AK (standardized Z-scores) in wild apple vigor classes across different months. Only soil factors with significant differences are presented. Different lowercase letters indicate significant differences (P < 0.05), TK, soil total potassium; AP, available phosphorus; AK, available potassium.

Month significantly affected TK (*P* < 0.001), AK (*P* < 0.001), and AP (*P* = 0.020) (**Table 1**). Over the seven-month sampling period (April–October), TK concentrations were lowest in June and July, with concentrations in October being significantly higher than those in June and July by 9.52% and 12.38%, respectively (**Figure 2**). TK gradually decreased from April to July and then increased from August to October. In October, AK was significantly higher than in April–September, with a 67.1% increase compared to September. Although AK did not differ significantly among months from April to September, it exhibited a fluctuating downward trend. AP in October was significantly higher than in June, showing an increase of 106.3%. In addition, a significant interaction between month and vigor class was observed only for TP. However, TP itself was not significantly influenced by month alone (**Table 1**).

### 2.2 Variation in SMF and TVN across vigor classes and growth periods

**Figure 3:**
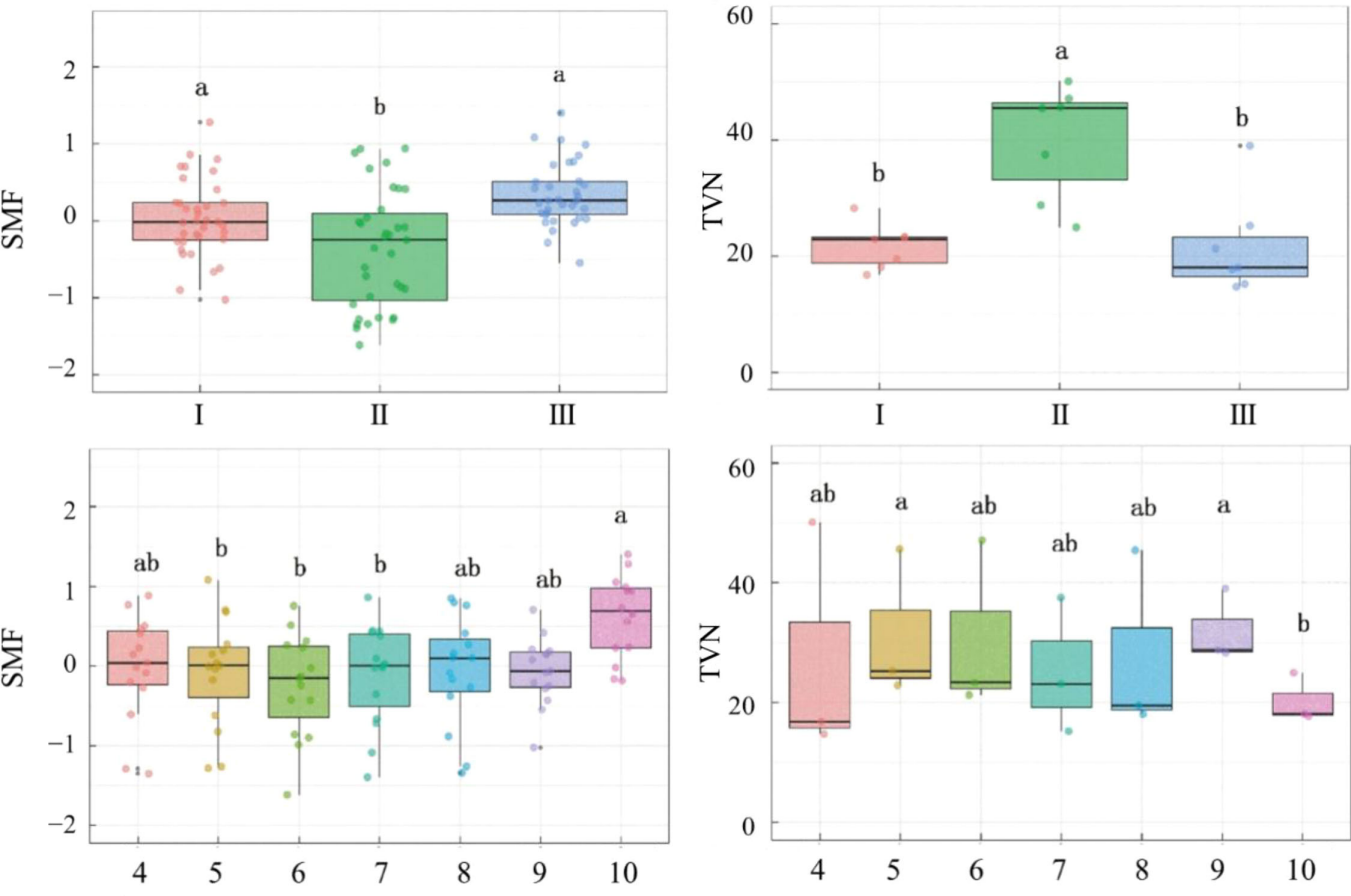
Differences in SMF and TVN (%) among wild apple vigor classes (I, II, and III) across months (April–October). Different lowercase letters indicate significant differences (P < 0.05).

Vigor class significantly influenced both SMF (*P* < 0.001) and TVN (*P* < 0.001) (**Table 2**). SMF in class II was significantly lower than that in both class I and class III (**Figure 3**), a pattern consistent with the trends observed for soil SOC, TN, TP, AN, AP, and AK. In contrast, TVN in class II was significantly higher than that in classes I and III, reflecting greater temporal variability in SOC, TN, TP, AN, AP, and AK under moderate degradation.

**Table 2:** Effects of growth period (month), vigor classes, and their interaction on SMF and TVN in wild apple vigor classes.

Resource of variation	SMF		TVN	
	*F*	*P*	*F*	*P*
Month	3.951	0.002	20.892	0.002
Vigor	15.654	<0.001	56.37	<0.001
Month × Vigor	0.678	0.767	—	—

Month also had a significant effect on SMF (*P* = 0.002) and TVN (*P* = 0.002) (**Table 2**). SMF in October was significantly higher than in all months from April to September (**Figure 3**), aligning with the seasonal increases observed in TK, AK, and AP. Conversely, TVN in October was significantly lower than in May and September, corresponding to the higher nutrient variability recorded during those months.

**Figure 4:**
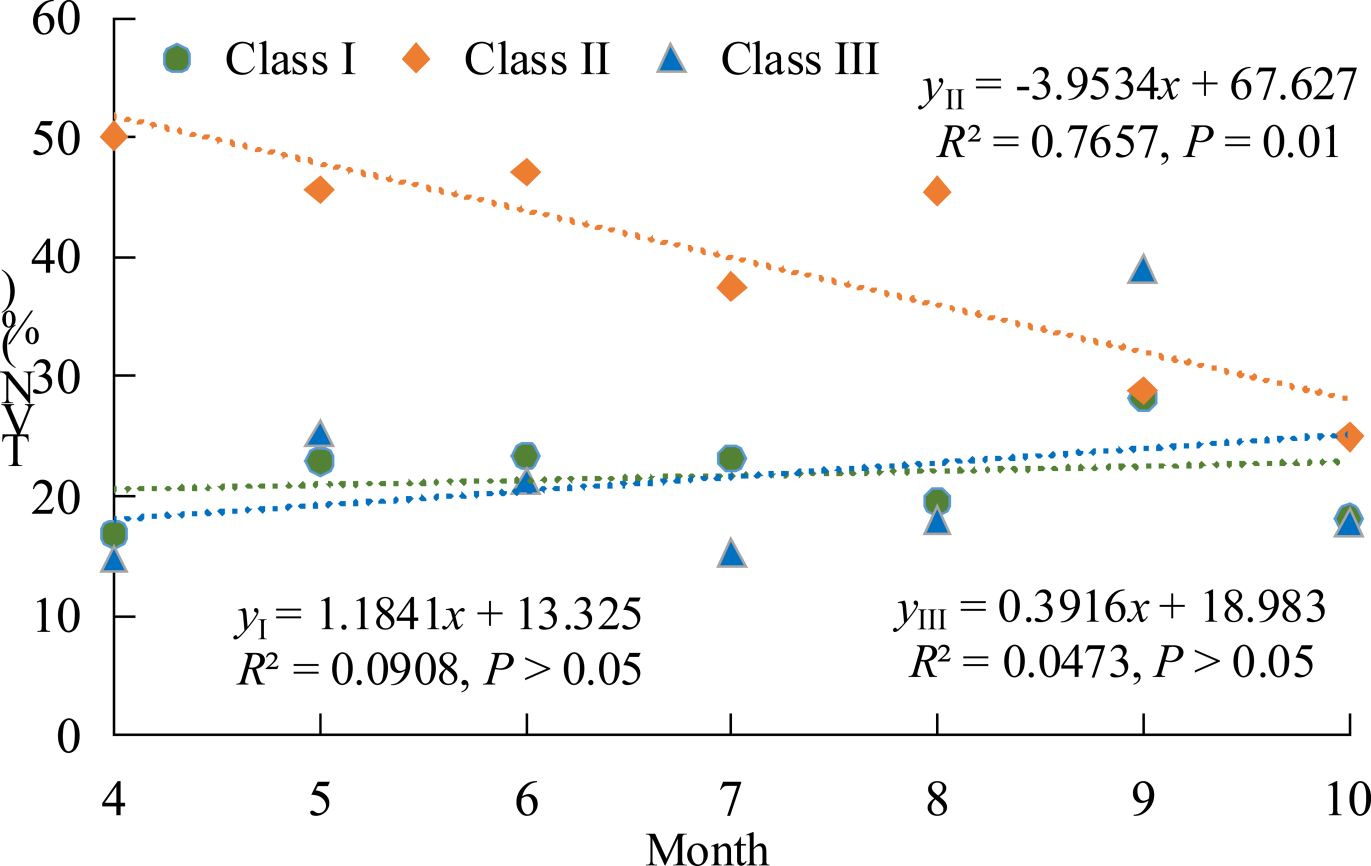
Monthly trends in TVN across wild apple vigor classes (I, II, and III).

Furthermore, TVN in vigor class II exhibited a significant declining trend over the growing season (*P* = 0.01), peaking during the early growth stage and returning to levels comparable to those of classes I and III by the late growth stage (**Figure 4**). By contrast, TVN in class I and class III showed no significant overall change throughout the growing season, suggesting that moderately degraded trees undergo ecological processes distinct from those of relatively healthy (class I) or severely degraded (class III) individuals.

### 2.3 Drivers of SMF and TVN across tree vigor classes

**Figure 5:**
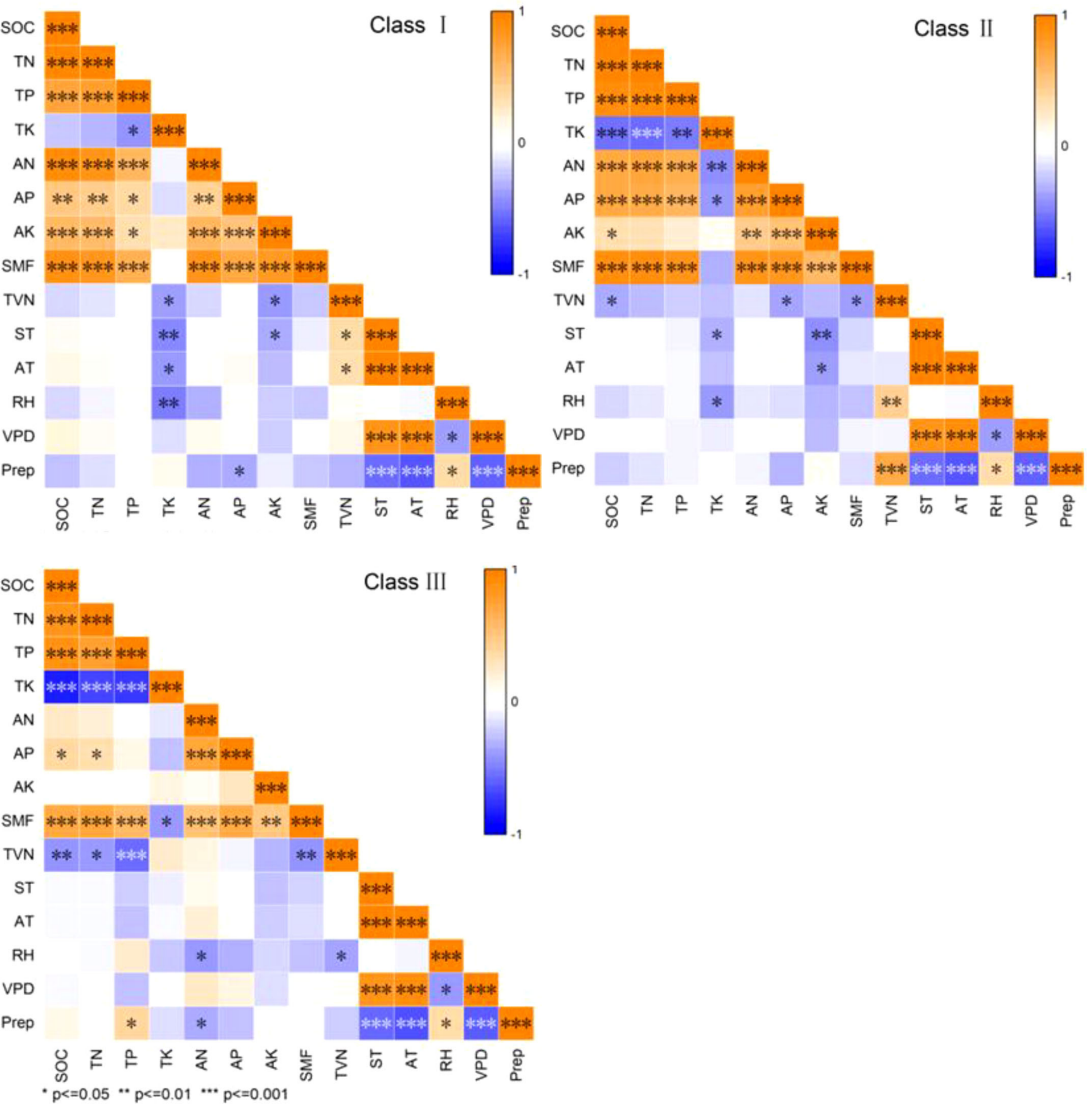
Correlations of single soil function (nutrient levels), SMF, TVN and meteorological factors in wild apple tree vigor classes under three vigor classes (I, II, and III). SOC, TN, TP, TK, AN, AP, AK, SMF, TVN, ST, AT, RH, VPD, and Prep represent soil organic carbon, total nitrogen, total phosphorus, total potassium, available nitrogen, available phosphorus, available potassium, soil multifunctionality, temporal variability of nutrients, mean monthly soil temperature, mean monthly air temperature, mean monthly relative humidity, mean monthly vapor pressure deficit, and monthly cumulative precipitation, respectively.

As shown in the correlation matrix (**Figure 5**), the relationships between soil properties, meteorological factors, and functional metrics varied across the vigor classes of *M. sieversii*. In vigor class I, TK was significantly negatively correlated with soil temperature, air temperature, and relative humidity; AP was significantly negatively correlated with precipitation; and AK was significantly negatively correlated with soil temperature. TVN was significantly positively correlated with both soil and air temperatures but was negatively correlated with TK and AK. In class II, TK was significantly negatively correlated with soil temperature and relative humidity, while AK showed significant negative correlations with soil temperature and air temperature. In contrast, TVN was significantly positively correlated with relative humidity and precipitation but was negatively correlated with SOC and AP. In class III, TK was significantly positively correlated with precipitation, whereas AN was significantly negatively correlated with relative humidity and precipitation. Furthermore, TVN exhibited significant negative correlations with relative humidity, SOC, TN, and TP. Across all three vigor classes, SMF was significantly positively correlated with SOC, TN, TP, AN, AP, and AK but showed no significant relationships with any meteorological variables. Conversely, TVN was influenced by a combination of soil and meteorological factors. Notably, SMF and TVN were significantly negatively correlated in classes II and III (but not in class I), indicating that higher SMF was associated with lower TVN. This pattern is consistent with the trends illustrated in **Figure 3**.

**Figure 6:**
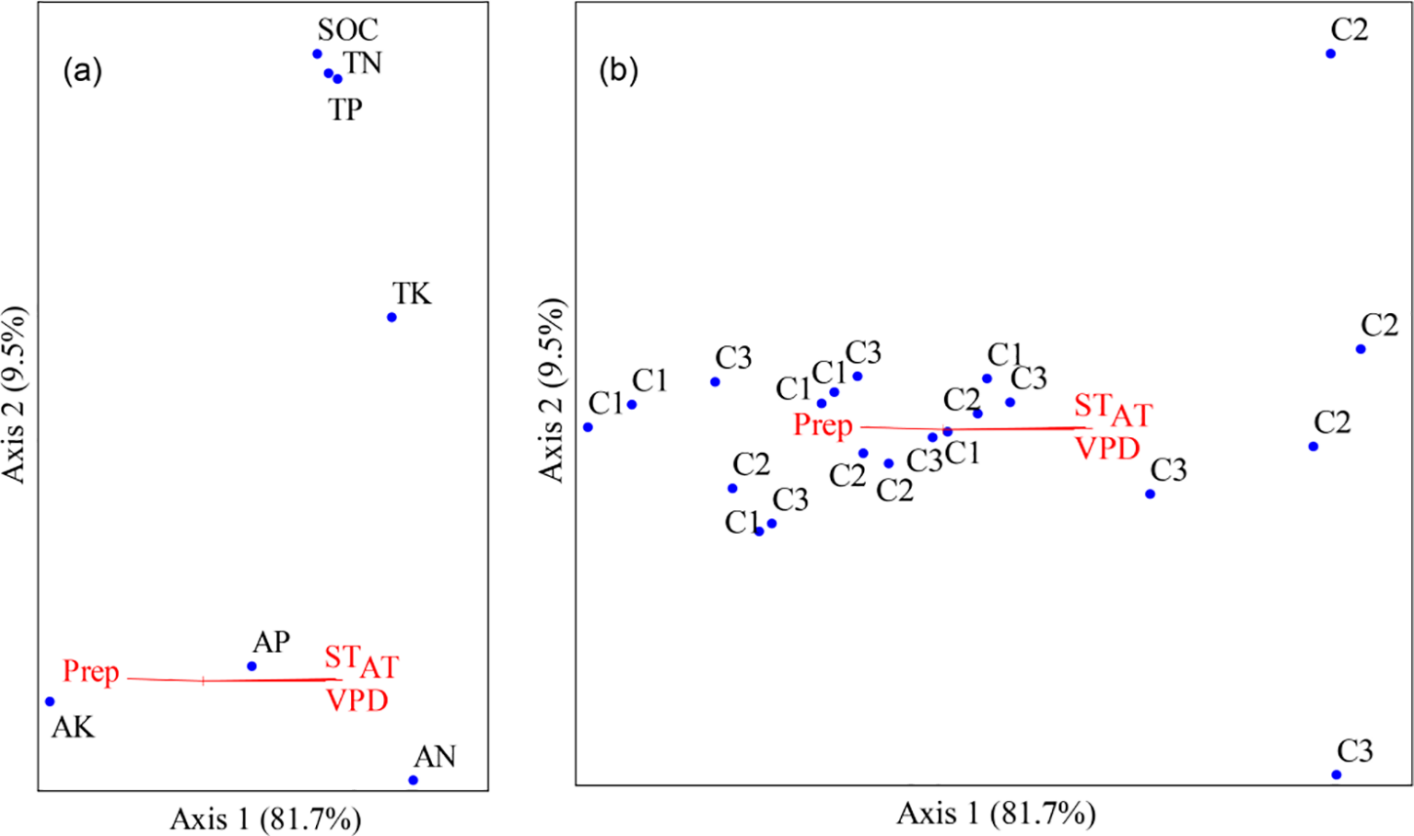
Non-metric multidimensional scaling (NMDS) ordination of soil functional variables in relation to meteorological factors across three vigor classes of wild apple (*Malus sieversii*) vigor classes.(C1, C2, and C3 corresponding to class I, II, and III, respectively). **(A)** Vectors represent meteorological variables Prep, AT, ST, RH, VPD, and points denote individual soil functional indicators SOC, TN, TP, TK, AN, AP, AK. The length and direction of vectors indicate the strength and nature of correlations with NMDS axes.C1, C2, and C3 corresponding to class I, II, and III, respectively. **(B)** Sample points are differentiated by vigor class: C1 (class I), C2 (class II), and C3 (class III). Axes 1 and 2 together explain 91.4% of the total variation (axis 1: 81.7%; axis 2: 9.5%). The spatial distribution of samples reveals a climate-driven gradient in soil nutrient composition, which underlies the differences in TVN and SMF among vigor classes.

Non-metric multidimensional scaling (NMDS) based on seven soil functional indicators revealed a discernible, though not fully discrete, ordination pattern in soil nutrient composition among vigor classes of *M. sieversii* (**Figure 6**). Axis 1 accounted for 81.7% of the total variation and was significantly correlated with several meteorological variables: air temperature (AT, *r* = 0.621), soil temperature (ST, *r* = 0.606), and vapor pressure deficit (VPD, *r* = 0.582) showed positive associations, whereas precipitation (Prep, *r* = −0.460) was negatively correlated (all *P* < 0.05; **Table 3**). In contrast, axis 2 explained only 9.5% of the variation and showed no significant correlation with any measured environmental variable. Soil nutrients exhibited differentiation along both ordination axes. Axis 1, which appeared to be primarily associated with climatic gradients, positioned AK toward the cool–humid end (left), whereas AN and AP were located toward the warm–dry end (right). In contrast, axis 2 showed no significant correlation with any measured meteorological variables; nevertheless, SOC, TN, TP, and TK clustered in the upper half of the ordination space, while AN, AP, and AK were concentrated in the lower half. Consistent with this interpretation, AK was strongly negatively correlated with axis 1 (*r* = −0.639), while AN exhibited the strongest association with axis 2 (*r* = −0.585), further indicating divergent responses among nutrient forms. When samples were grouped by tree vigor class (C1, C2, and C3), considerable overlap was observed in the ordination space, implying that similar soil functional states may occur across different vigor classes. Nevertheless, a tentative spatial trend emerged: C1 samples were predominantly located on the left (cool–humid end), some C2 samples extended toward the right (warm–dry end), and C3 samples were largely concentrated in the central region. This pattern may reflect differences in the micro-environmental contexts associated with each vigor class. Intriguingly, C2 samples, which are often situated in regions exhibiting greater climatic variability along axis 1, also tend to exhibit higher TVN and lower SMF (**Figure 3**).While these observations are consistent with the hypothesis that climate-driven shifts in soil nutrient composition contribute to the trade-off between stability (low TVN) and functionality (high SMF), such an interpretation remains provisional and warrants further examination through complementary analytical approaches, such as partial least squares path modeling (PLS-PM).

**Table 3:** Correlation coefficients between soil function parameters and meteorological factors of across three wild apple tree vigor classes and the first two NMDS axes.

Axis	Soil functional parameter							Meteorological factor				
	SOC	TN	TP	TK	AN	AP	AK	ST	AT	RH	VPD	Prep
Axis 1	−0.226	−0.223	−0.324	−0.229	−0.007	−0.232	−0.639**	0.606**	0.621**	−0.086	0.582**	−0.460*
Axis 2	0.253	0.255	0.400	−0.264	−0.585**	−0.303	−0.409	0.079	0.040	0.203	−0.021	0.071

*p < 0.05, **p < 0.01, indicating levels of statistical significance.

### 2.4 Causal pathways influencing SMF and TVN

**Figure 7:**
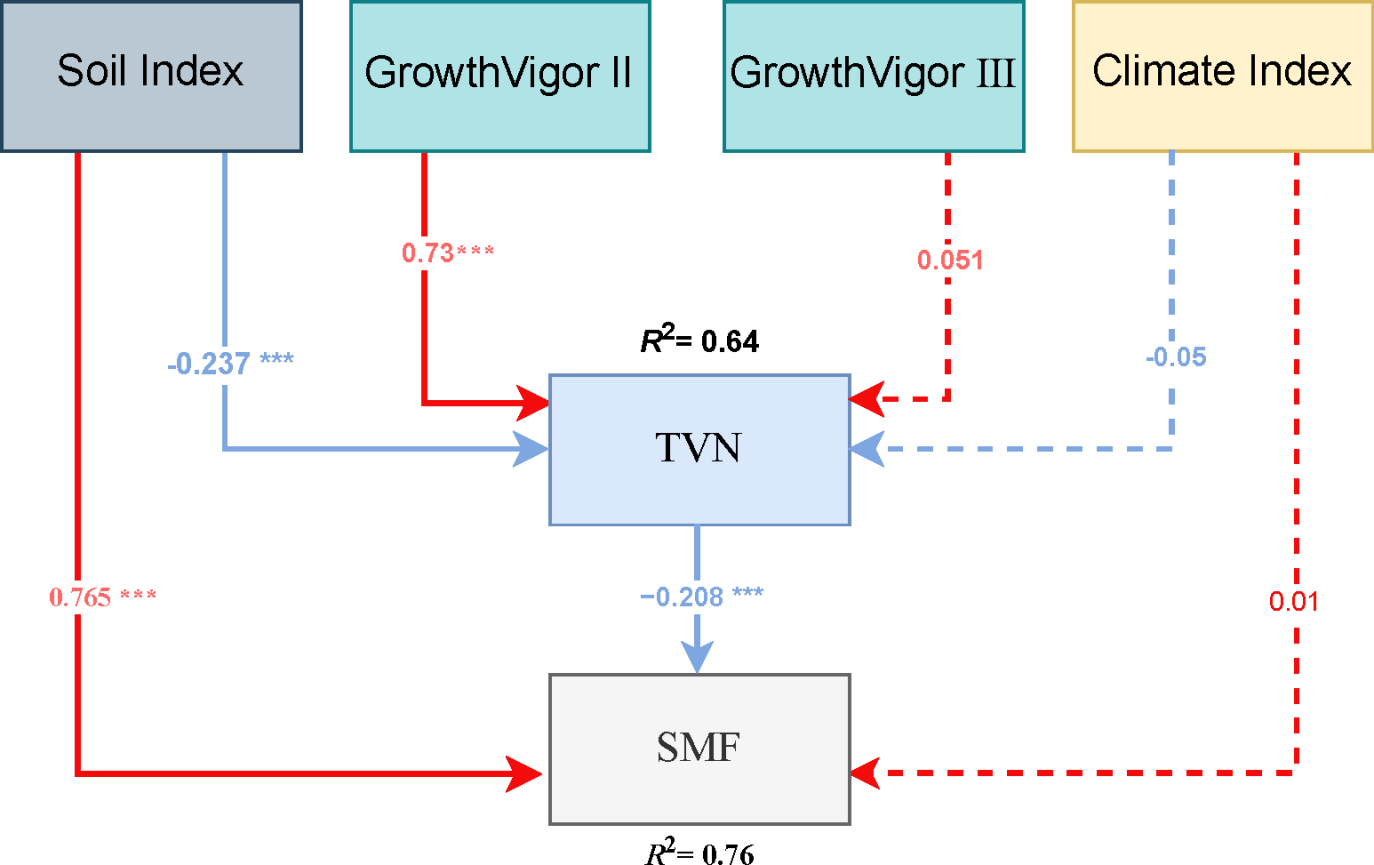
Partial least squares path model analyzing the effects of vigor classes, meteorological factors, and soil factors on SMF and TVN in wild apple tree vigor classes. *p < 0.05, **p < 0.01, ***p < 0.001.

In this study, we employed partial least squares path modeling (PLS-PM) to analyze the causal pathways through which various factors influence soil multifunctionality (SMF) and temporal variability of soil nutrients (TVN). Based on multicollinearity diagnostics and factor loading analyses, composite indices were constructed for soil nutrients (SOC + TN + TP) and climate (air temperature - precipitation, AT - Prep). All latent variables were measured with single indicators, ensuring statistical robustness (average variance extracted, AVE = 1.0). The model explained 75.9% and 63.8% of the variance in SMF and TVN, respectively (**Figure 7**). A significant negative direct effect of TVN on SMF was detected (path coefficient = –0.208, *P* < 0.001), indicating that reduced temporal stability in nutrient supply directly undermines soil multifunctionality.

#### 2.4.1 Soil factors: core pathways governing SMF

The soil nutrient composite index exhibited the strongest positive direct effect on SMF (path coefficient = 0.765, *P* < 0.001), suggesting that the accumulation of SOC, TN, and TP enhances functional redundancy and microbial metabolic capacity within the soil ecosystem, thereby increasing SMF. Simultaneously, the soil nutrient index showed a significant negative effect on TVN (path coefficient = –0.237, *P* < 0.001), indicating that nutrient-rich soils exhibit greater temporal stability during the growing season. Specifically, soils with higher nutrient content are likely able to sustain continuous microbial cycling and more uniform litter decomposition, thereby reducing seasonal fluctuations in nutrient availability.

#### 2.4.2 The critical role of wild apple tree growth vigor

The model revealed that moderately degraded (Class II) growth vigor had a significant positive effect on TVN (path coefficient = 0.730, *P* < 0.001), whereas severely degraded (Class III) growth vigor showed no significant effect (*P* = 0.435). This indicates that temporal variability in soil nutrients (TVN) is significantly elevated under Class II conditions.

**Figure 8:**
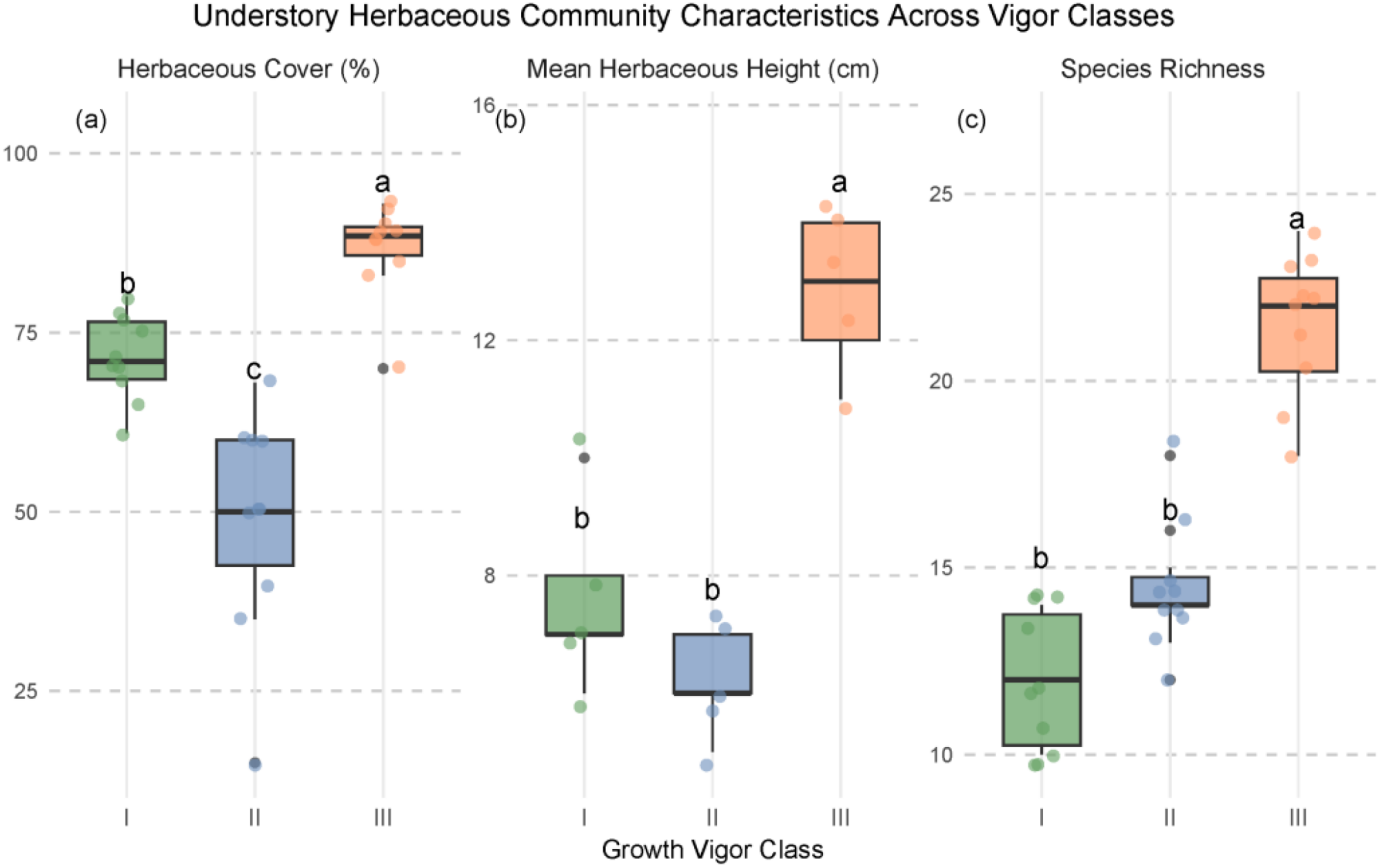
Understory herbaceous community characteristics across tree vigor classes at the end of the growing season. Shown are (a) herbaceous cover (%), (b) mean herbaceous height (cm; September only), and (c) species richness (mean number of species per plot). Boxes represent interquartile ranges (IQR), horizontal lines indicate medians, whiskers extend to 1.5 × IQR, and individual points show raw observations (n = 10 plots per class). Different lowercase letters above boxes denote significant differences among vigor classes (one-way ANOVA followed by Tukey’s HSD test, *P* < 0.05). Class III (severely degraded) exhibits significantly higher herbaceous cover and species richness compared to Classes I (relatively healthy) and II (moderately degraded), suggesting enhanced understory colonization under open canopy conditions.

Understory vegetation data provide important community-level support for interpreting these PLS-PM results (**Figure 8**): herbaceous cover was lowest in Class II (label c), intermediate in Class I (b), and highest in Class III (a), with all three classes significantly different from one another. Both herbaceous height (measured in September) and species richness were significantly highest in Class III (a), while Classes I and II did not differ significantly (both labeled b). Collectively, these observations suggest that tree growth vigor influences temporal nutrient dynamics by modulating understory vegetation structure. Specifically, under Class II vigor, the markedly reduced herbaceous cover, coupled with lower stature and species richness, likely reflects insufficient light availability for robust understory development despite partial canopy opening. This may weaken buffering of microclimatic fluctuations and reduce organic matter inputs, thereby exacerbating seasonal imbalances in nutrient supply and demand and ultimately driving the observed increase in TVN.

#### 2.4.3 Effects of climatic factors

Contrary to expectations from the NMDS ordination (which showed strong correlations between axis 1 and meteorological variables), the PLS-PM analysis revealed no significant direct effects of the climate composite index on either SMF (path coefficient = 0.012, *P* = 0.813) or TVN (path coefficient = –0.053, *P* = 0.381). This index was defined such that positive values represent warm–dry conditions and negative values indicate cool–humid conditions. This suggests that, across the sampled sites in the Ili Valley, our analysis did not detect a statistically significant direct influence of the regional climate gradient on SMF or TVN after controlling for tree vigor and soil fertility. While climate may shape broad-scale patterns of vegetation and soil development, our results highlight that fine-scale drivers, particularly tree health status and soil nutrient capital, are more immediate determinants of ecosystem functional outcomes in degraded wild apple forests. This underscores the importance of targeted, site-level conservation strategies over generalized climate-adaptation approaches in this system.

## 3 Discussion

### 3.1 Class II trees exhibit a critical loss of both function and stability

Our results indicate that SMF is lowest in *M. sieversii* trees of moderate degradation (growth vigor Class II), while TVN reaches its highest level in the same class. SMF reflects the integrated capacity of soils to sustain both long-term nutrient pools and short-term nutrient supply, whereas TVN uses spatial heterogeneity among trees within a vigor class as a proxy for temporal instability under reduced functional redundancy (Yachi and Loreau, 1999). In contrast, both relatively healthy trees (Class I) and severely degraded trees (Class III) maintain comparatively higher SMF, suggesting a non-monotonic relationship between tree vigor decline and soil functional stability. This pattern suggests that moderate degradation may represent a critical transitional phase, during which ecosystem stability is disproportionately compromised relative to structural deterioration and may potentially precede overt structural collapse.

### 3.2 Climate indirectly promotes functional instability by favoring vulnerable degradation classes

The latent variables employed in the PLS-PM are grounded in ecological theory. The climate composite index was constructed such that higher values correspond to warmer and drier conditions, a formulation consistent with the energy–water co-limitation framework that underpins vegetation and ecosystem dynamics in water-limited regions (Stephenson, 1998; Schwinning et al., 2004). Specifically, we assigned a positive weight to standardized mean air temperature and a negative weight to standardized total precipitation during the growing season. This formulation aligns closely with the primary environmental gradient captured by NMDS axis 1. Similarly, the soil nutrient index, based on SOC, TN, and TP, forms a distinct cluster in the ordination space.

Despite the strong association between climatic conditions and site distribution along the NMDS gradient, the PLS-PM analysis revealed no statistically significant direct effect of the climate index on either SMF (*β* = 0.012, *P* = 0.813) or TVN (*β* = –0.053, *P* = 0.381). A key methodological consideration is that tree growth vigor classes (I–III) were derived from field-based visual assessments of branch dieback rates and represent discrete degradation classes rather than continuous, climate-driven physiological responses (Tao et al., 2020); thus, they were treated as exogenous predictors in the model. Because tree vigor classes were treated as exogenous predictors (rather than endogenous latent variables), the PLS-PM framework does not estimate indirect effects of climate mediated through *M. sieversii* growth vigor. Instead, the convergence of NMDS and PLS-PM results reveals a consistent spatial pattern: warmer–drier sites are predominantly occupied by Class II *Malus sieversii* trees, which in turn show the strongest positive association with TVN (*β* = 0.730, *P* < 0.001). This co-occurrence suggests that regional climate may influence soil functional stability not through direct abiotic forcing, but by promoting the prevalence of specific *M. sieversii* vigor classes characterized by moderate degradation, which is consistent with global dryland studies showing that climate shapes ecosystem function primarily through its influence on vegetation structure and condition (Maestre et al., 2012). Importantly, our model identifies tree growth vigor as a key mediator between environmental stress and soil dynamics. Moderate to severe degradation of *M. sieversii* classes, characterized by partial canopy dieback and reduced photosynthetic capacity, leads to higher temporal variability in soil nutrients (TVN), which in turn suppresses soil multifunctionality (SMF). This suggests that functional decline is driven more by the condition of the dominant tree species (*M. sieversii*) than by direct climatic forcing, highlighting the value of monitoring wild apple health as an early indicator of ecosystem vulnerability.

### 3.3 Understory compensation mitigates functional loss under severe degradation

The heightened vulnerability of Class II likely stems from an imbalance between nutrient supply and demand under transitional canopy conditions. Field surveys show that herbaceous cover is lowest in Class II (**Figure 8**), implying reduced inputs of litter and root exudates. Concurrently, sparse canopy closure may amplify microclimatic fluctuations, disrupting microbial activity and nutrient cycling rhythms, and leading to erratic nutrient availability and elevated TVN. Higher TVN reflects greater intra-seasonal variability in nutrient supply, which may hinder the simultaneous operation of multiple, often competing, soil processes (Wagg et al., 2014; Griffiths and Philippot, 2013).

In contrast, despite severe tree decline, Class III plots exhibit significantly higher herbaceous species richness and ground cover (**Figure 8**). Diverse understory communities can enhance functional redundancy through complementary resource use, stabilize organic matter inputs via mixed litter decomposition, and sustain rhizosphere activity even in the absence of vigorous overstory trees (Dijkstra et al., 2013; Landuyt et al., 2020). These mechanisms may buffer against further increases in TVN and help preserve core soil functions. Additionally, frequent signs of livestock grazing beneath Class III trees suggest that biotic disturbances might interact with this compensatory process, for example, by enhancing nutrient inputs through dung deposition or altering plant community composition via selective herbivory. However, as grazing intensity was not quantified in this study, the net effect of such interactions remains uncertain and warrants further investigation.

### 3.4 Implications of TVN as an early-warning indicator under climate change

The strong negative correlation between SMF and TVN, particularly pronounced in Class II, suggests that temporal nutrient variability may serve as an early-warning indicator of ecosystem degradation. High temporal variability typically reflects a loss of functional buffering capacity and often precedes irreversible declines in mean functionality (Ding and Eldridge, 2021). This aligns with theory on critical transitions in complex systems, where increased variance in state variables is a hallmark of declining resilience and proximity to a tipping point (Scheffer et al., 2009). Projected increases in temperature and shifts in precipitation patterns may exacerbate nutrient leaching, disrupt microbial metabolic balance, or desynchronize phenological cycles of litter decomposition, all of which can erode the temporal coherence of soil functions (Huntington, 2006; Chen et al., 2024). Our findings are consistent with a broader paradigm in dryland ecology: moderate disturbance often maximizes functional instability, whereas extreme degradation may paradoxically lead to relative stabilization through system homogenization or compensatory colonization (Berdugo et al., 2020; Wu et al., 2024). From a conservation perspective, this implies that Class II wild apple trees, although not yet exhibiting obvious structural collapse, may already be approaching a stability threshold. Urgent interventions, such as targeted nutrient supplementation or understory restoration, could help mitigate further degradation and potentially avert irreversible ecosystem decline (Yan et al., 2021).

This study has a key methodological limitation that warrants consideration. All observations were conducted within a single, spatially restricted plot (40 × 60 m) characterized by relatively homogeneous environmental conditions. Although this design effectively minimizes large-scale environmental variation and enables a focused assessment of how tree vigor status influences soil multifunctionality (SMF) and temporal variability of soil nutrients (TVN), it also means that samples across vigor classes were not fully spatially independent. Unmeasured microenvironmental gradients or legacy effects within the plot, such as historical minor disturbances or localized differences in hydrothermal conditions, may still influence soil nutrient patterns and functional processes. Consequently, the statistical inferences presented here are best interpreted as a case study of this specific local ecosystem. Broader generalizability across larger spatial scales or diverse geographic regions will require validation through future multi-site and multiscale investigations.

## 4 Materials and methods

### 4.1 Study area

The study was conducted in the Ili River Valley of the western Tianshan Mountains and the western mountainous regions of the Junggar Basin in Xinjiang. The Ili River Valley constitutes a core distribution zone for *M. sieversii*, occurring predominantly mid- and low-elevation zones (1,000–1,800 m) along the Ili River, typically in a fragmented, insular pattern (Yan and Xu, 2010, pp. 10–20). The region experiences a temperate continental climate with pronounced oceanic influences, characterized by a mean annual precipitation of 416 mm, mean annual temperature of 7–9°C, approximately 150 days of snow cover, and an annual sunshine duration of approximately 2,500 hours. An inversion layer commonly forms between 800 and 1,600 m elevation, buffering vegetation from cold air incursions and facilitating the development of forest communities resembling oceanic deciduous broadleaf forests. Within these forests, *M. sieversii* occurs either in pure stands or in mixed associations with *Armeniaca vulgaris* var. *ansu*, *Juglans regia*, and *Betula tianschanica* (Yan and Xu, 2010, pp. 10–20; Zhang et al., 2023). Soils under *M. sieversii* forests in the Ili region predominantly develop on loess-like parent materials, exhibiting a thick humus horizon, high porosity, loose structure, and enrichment in silicate minerals and base cations, which collectively contribute to relatively high soil fertility (Tao et al., 2016).

### 4.2 Selection of wild apple trees with different vigor classes

**Figure 9:**
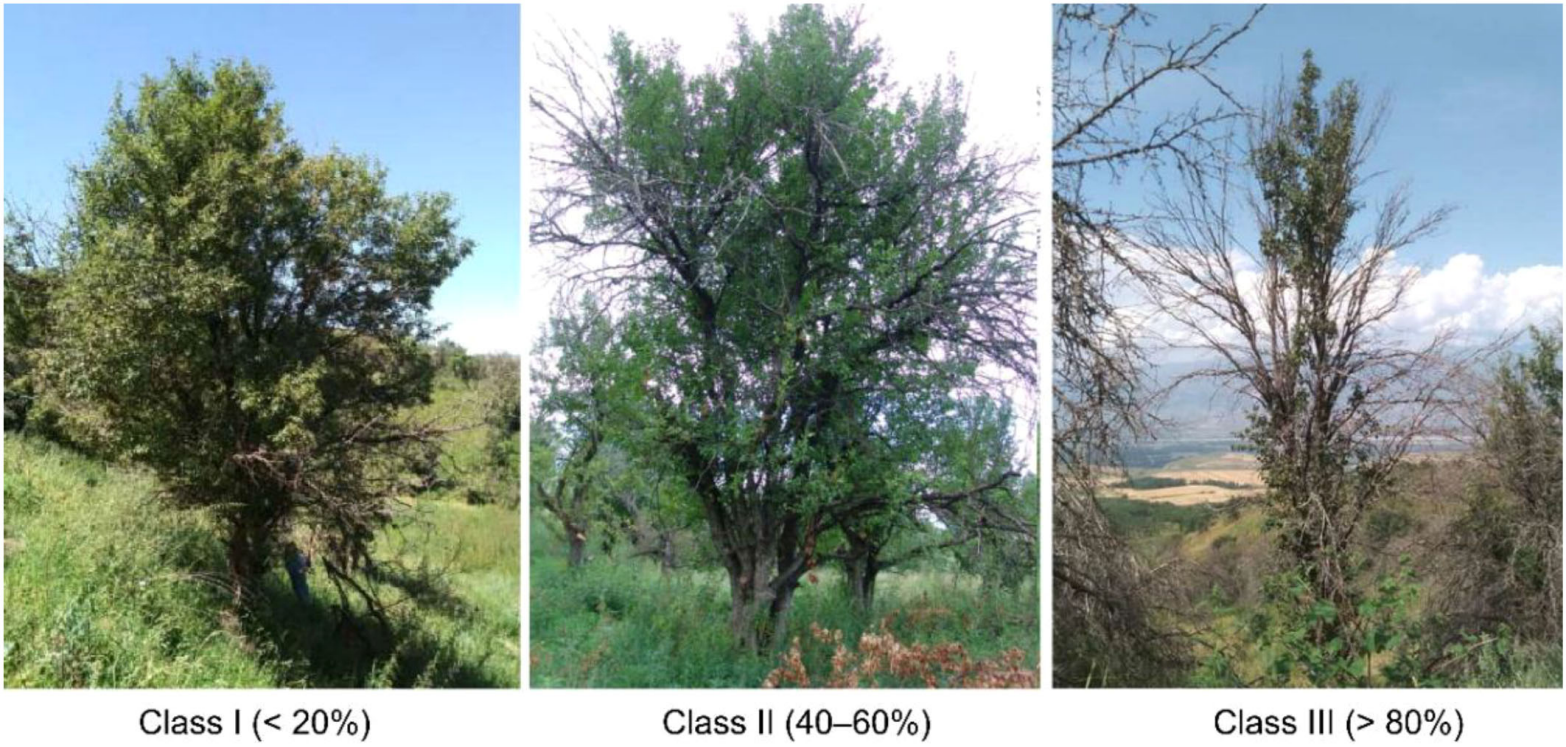
Morphology of *M. sieversii* with different vigor classes (cited from Tao et al., 2020).

To minimize background soil variability, three vigor classes of *M. sieversii* were selected within a fixed plot (40 m × 60 m; with the long side oriented horizontally, the plot was unfenced) to represent tree growth status (**Figure 9**). The vigor classes were classified as follows: Class I (relatively healthy, with a dead branch percentage < 20%), Class II (moderately degraded, dead branch percentage 40–60%), and Class III (severely degraded, dead branch percentage > 80%). The dead branch ratio was calculated as the percentage of dead branches relative to the total number of branches per tree (Tao et al., 2020). Due to the extreme scarcity of healthy *M. sieversii* individuals in Xinyuan County, Ili, it was not possible to select a sufficient number of such trees within the plot; consequently, this study did not include a fully healthy, zero-dead-branch group as a control. For each vigor class, 10 *M. sieversii* individuals of similar size (diameter at breast height [DBH] 25–30 cm) were selected as replicates and marked with colored ribbons in three different colors, resulting in a total of 30 individuals. To minimize potential interactions in soil water and nutrient uptake, a minimum distance of 4 m was maintained between adjacent trees.

### 4.3 Understory herbaceous survey

In September and October 2018, standardized understory herbaceous surveys were conducted beneath each of the 30 marked *M. sieversii* trees (10 per vigor class). Within a 1 m × 1 m quadrat placed randomly within a 1-m radius around each tree base, we recorded: (1) herbaceous species richness (2) visual estimation of total herbaceous cover (%), and (3) mean herbaceous height (cm), measured as the average height of the five tallest non-woody plants. Height data were only collected in September to capture peak growing conditions. All quadrats were positioned to avoid direct overlap with soil coring locations used in 2018.

### 4.4 Soil sample collection and analysis

From April to October 2018, soil samples were collected monthly on the 20th from all 30 marked *M. sieversii* trees (10 per vigor class). Using a 10-cm inner-diameter soil corer, surface soil (0–10 cm) was sampled within a 1-m radius around the base of each *M. sieversii* tree. Care was taken to avoid resampling the same points in different months. The collected samples represent bulk surface soil influenced by tree activity but were not separated into rhizosphere and non-rhizosphere fractions. After removing surface organic matter and fine roots, soil samples were air-dried in the shade. The naturally dried soils were then sieved through a 2-mm mesh. Then, soil organic carbon (SOC, g kg^-1^), total nitrogen (TN, g kg^-1^), total phosphorus (TP, g kg^-1^), total potassium (TK, g kg^-1^), soil available nitrogen (AN, mg kg^-1^), available phosphorus (AP, mg kg^-1^), and soil available potassium (AK, mg kg^-1^) contents were determined by standard methods referenced from *Soil Agricultural Chemical Analysis* (Bao, 2000, pp. 8–28). All soil nutrient concentrations are reported on an air-dry mass basis.

### 4.5 Collection of meteorological factors

Daily meteorological data were obtained from a long-term monitoring plot of the wild fruit forest in the Ili Botanical Garden, located approximately 300 m west of the study area. From these data, monthly averages of soil temperature (ST), vapor pressure deficit (VPD), air temperature (AT), relative humidity (RH), and cumulative monthly precipitation (Prep) were extracted.

### 4.6 Ecosystem functional metrics

#### 4.6.1 Soil multifunctionality

Soil multifunctionality (SMF) is based on seven soil nutrient indicators: SOC, TN, TP, TK, AN, AP, and AK. These indicators collectively represent two distinct functional aspects of soil nutrient dynamics: (1) stable nutrient pools (SOC, TN, TP, TK), which reflect long-term fertility and organic matter storage; and (2) plant-available nutrients (AN, AP, AK), which indicate short-term nutrient supply capacity. Because these dimensions underpin key processes such as nutrient provisioning and support for biological activity, SMF—derived from these complementary metrics—is closely linked to nutrient cycling in arid ecosystems and can effectively reflect multiple ecosystem functions, including nutrient supply, water retention (mediated through soil organic matter), and the capacity to sustain diverse life processes. The selection of these complementary indicators aligns with recent recommendations emphasizing that ecosystem multifunctionality assessments should integrate distinct functional dimensions rather than redundant metrics. There are various methods to quantify SMF, each with its own advantages and limitations. For instance, the factor analysis approach requires clustering, dimensionality reduction, and factor extraction of functional indices, with the resulting factor scores representing multifunctionality. In contrast, the mean-based method (Z-scores) is the most commonly used, representing ecosystem multifunctionality by calculating the average standardized scores across different ecosystem functions. First, the standardized scores of seven individual soil nutrient indicators were calculated. The mean of these standardized scores was then computed to obtain the SMF for a given month in a specific *M. sieversii* habitat. Numerous studies have demonstrated that SMF values derived from these two methods are highly and significantly positively correlated, indicating that they are largely interchangeable (Zhang et al., 2022b). Therefore, the mean-based method was selected for SMF analysis in this study.

#### 4.6.2 Temporal variability of nutrients

To evaluate the temporal stability of soil nutrient supply across growth vigor classes during the growing season, we developed an indirect indicator termed the Temporal Variability of Soil Nutrients (TVN). This metric was formulated in response to the highly fragmented spatial distribution of wild apple (*M. sieversii*) populations, which precludes the establishment of replicated plots across natural degradation gradients. Although our dataset comprises monthly soil collections from April to October, our objective was not to characterize temporal fluctuations at the level of individual trees. Instead, we aimed to assess the collective dynamic stability of soil nutrient availability for each growth vigor class on a monthly basis. Standard time series metrics, such as the temporal standard deviation computed for single trees, would reflect only within-individual variability and fail to capture the emergent stability properties of the class as a functional unit.

Accordingly, we implemented an alternative framework grounded in ecological stability theory, wherein spatial heterogeneity among replicates within a group at a given point in time serves as a proxy for its temporal instability. Building on the insurance hypothesis (Yachi and Loreau, 1999), which posits that greater functional or response diversity enhances a system’s capacity to maintain stable functioning under environmental perturbations, we reasoned that higher spatial variation in soil nutrients among replicate trees within the same vigor class and month indicates reduced functional redundancy and weaker buffering capacity. Consequently, such variation is interpreted as reflecting greater expected temporal instability in nutrient supply over the growing season. This interpretation aligns with the conceptual framework of Arnoldi et al. (2016), who rigorously define variability as the magnitude of fluctuations in system state under persistent environmental stochasticity and identify it as a fundamental and quantifiable dimension of ecological stability, distinct from resilience and reactivity. Empirical and theoretical work further supports that systems exhibiting high intra-group variability often lack the compensatory dynamics necessary for functional stability (Isbell et al., 2015).

Specifically, for each combination of growth vigor class and month:

1. We compiled all soil nutrient measurements from replicate trees within that class and month, forming a spatial dataset.
2. For each of the seven soil indicators (SOC, TN, TP, TK, AN, AP, AK), we calculated the coefficient of variation (CV):

$$CV_{j}=\frac{\mu_{j}}{\sigma_{j}}\times 100\%$$

where *σ_j_* and *μ_j_* are the standard deviation and mean of the j-th nutrient across all replicate trees in that class-month unit. The CV is dimensionless and expresses relative variability, enabling comparison across variables with different units (e.g., g/kg vs. mg/kg).

3. We then computed TVN as the arithmetic mean of the seven individual CVs:


$$TVN=\frac{1}{7}\sum_{j=1}^{7}CV_{j}$$

To our knowledge, this specific formulation of TVN as a composite index that averages the coefficients of variation of multiple soil nutrients to infer temporal instability from cross-sectional spatial heterogeneity has not been previously reported in the soil or ecological literature. While the use of CV to quantify relative variability is well established (Legendre and Legendre, 2012), and variability itself is widely recognized as a key aspect of ecological stability (Arnoldi et al., 2016), our approach represents a pragmatic adaptation for systems where longitudinal replication is constrained by spatial fragmentation and logistical limitations.

It should be noted that while TVN incorporates all seven nutrients to ensure ecological comprehensiveness, the subsequent partial least squares path modeling (PLS-PM) analysis employed a composite soil fertility index derived solely from SOC, TN, and TP. These three indicators were selected based on statistical diagnostics, including condition indices, exploratory factor loadings, and PLS outer model weights, to enhance model parsimony, identifiability, and predictive performance.

### 4.7 Descriptive statistics and univariate analyses

Descriptive statistical analyses and normality tests (Kolmogorov–Smirnov test) were performed on the soil nutrient data collected from *M. sieversii* trees of different vigor classes. It should be noted that, for consistency with SMF, individual soil functions were represented by standardized scores obtained using the mean-based Z-score method (ranging from −2 to 2), which were fully consistent with the results from the original data statistical analyses (Zhang et al., 2022b). A two-way ANOVA within the framework of a general linear model (GLM) was used to examine the effects of month and vigor class on individual soil functions, SMF, and TVN, as well as their interactive effects. One-way ANOVA was used to compare differences in soil nutrients, SMF, and TVN among different months and vigor classes. Multiple comparisons were performed using Duncan’s test at a significance class of α = 0.05. Pearson correlation analysis was performed to examine the relationships between soil nutrients and meteorological factors, aiming to identify the main factors influencing SMF.NMDS was further employed to explore the relationships between the soil nutrient matrix and meteorological factors.

### 4.8 Partial least squares path modeling

#### 4.8.1 Latent variable construction and indicator selection

Prior to constructing the Partial Least Squares Path Model (PLS-PM), we conducted indicator screening and optimization based on prior correlation analyses and NMDS ordination results to ensure statistical robustness and mitigate multicollinearity issues. The procedures are detailed as follows:

1. Soil Nutrient Indicator Screening

To construct robust latent indicators and mitigate multicollinearity, we conducted a systematic diagnostic assessment of the seven initial soil nutrient variables (SOC, TN, TP, TK, AN, AP, AK). First, the condition number, defined as the ratio of the largest to smallest eigenvalue (λ_1_/λ_7_) derived from the correlation matrix of standardized variables, was calculated as 79.02, far exceeding the conventional threshold of 30 (Hair et al., 2010), indicating severe multicollinearity. We then performed an exploratory factor analysis (EFA) using principal axis factoring with a single factor extracted and no rotation. The results showed that SOC, TN, and TP exhibited high loadings on the dominant factor (0.975, 0.948, and 0.879, respectively), whereas TK (−0.488) and AK (0.412) had absolute loadings below the commonly used cutoff of 0.5, suggesting limited representation of the core soil functional dimension. Furthermore, the outer model of a full-indicator PLS-PM revealed that TK had a near-zero weight (−0.017) and a low loading (−0.422), reinforcing its redundancy. Guided by this convergent statistical evidence, we constructed a composite soil nutrient index using SOC, TN, and TP, which represent the central components of carbon, nitrogen, and phosphorus biogeochemical cycles and are universally recognized as foundational to soil fertility and ecosystem functioning (Delgado-Baquerizo et al., 2016).The composite index was constructed as the sum of Z-score standardized values, following standard practice for formative latent variable specification in PLS-PM (Chin, 2010):


Soil Index=Z(SOC)+Z(TN)+Z(TP), where Z denotes Z-score standardization.

2. Climate Variable Optimization

Although monthly mean air temperature (AT) and cumulative monthly precipitation (Prep) hold clear ecological relevance, incorporating them as separate indicators resulted in suboptimal measurement model quality (AVE = 0.433). To resolve this, we synthesized them into a single climate composite index:


Climate Index=Z(AT)–Z(Prep)

where Z denotes Z-score standardization. This formulation is ecologically grounded in the principle that evaporative demand (driven by temperature) and water supply (determined by precipitation) jointly regulate climatic water stress, a concept that is central to ecohydrological theory (Stephenson, 1998). Stephenson (1998) demonstrated that indices integrating these opposing processes, such as water deficit, better reflect the environmental stress experienced by plants than single variables or additive combinations of moisture-related scalars. In our index, positive values represent “warm–dry” conditions (high evaporative demand coupled with low water supply), indicating high hydrothermal stress, whereas negative values reflect “cool–humid” conditions (low demand and ample supply), indicating low stress. By subtracting standardized precipitation from standardized temperature, the directional effects of the two stressors are aligned, yielding a parsimonious yet ecologically interpretable metric well-suited for arid and semi-arid ecosystems.

3. Treatment of Tree Vigor Status

Tree vigor status, as a categorical variable, was encoded using dummy variables: Growth Vigor II (moderate decline, relative to Class I) and Growth Vigor III (severe decline, relative to Class I), with healthy trees (Class I) serving as the reference group. Through these optimizations, the Average Variance Extracted (AVE) for all latent constructs reached 1.0, effectively eliminating multicollinearity and ensuring the reliability and interpretability of subsequent model outputs.

#### 4.8.2 Model specification and validation

Building upon the optimized indicator set and composite indices, we constructed a PLS-PM using the “plspm” package in R version 4.2.2 to explicitly quantify the pathways and strengths through which environmental factors influence SMF and TVN in wild apple vigor classes, following established guidelines for PLS-PM specification, estimation, and validation (Chin, 2010). The final structural model comprised six latent variables: Climate, Soil, Growth Vigor II, Growth Vigor III, Temporal Variability of Nutrients (TVN), and Soil Multifunctionality (SMF). Path coefficients were tested for statistical significance via bootstrap resampling with 200 replications. Only statistically significant paths (p < 0.05) were retained in the final model. Normalized path coefficients (positive or negative) were used to represent directional causal relationships, and R² values were reported to indicate the proportion of variance explained in each endogenous latent variable.

All descriptive statistics, correlation analyses, and ANOVA tests were performed using SPSS 19.0. Non-metric multidimensional scaling (NMDS) was conducted using PC-ORD v5.0. Routine data processing and calculations were carried out in Microsoft Excel 2013, and all figures were generated using Origin 2019.

## 5 Conclusions

This study demonstrates that tree vigor status and intrinsic soil properties, in conjunction with regional climatic context, collectively shape patterns of soil nutrient composition, multifunctionality (SMF), and temporal stability, which we quantify as the Temporal Variability of Nutrients (TVN), in declining *M. sieversii* forests. NMDS revealed a dominant spatial gradient in soil nutrients aligned with climate, ranging from cool–humid to warm–dry conditions: labile forms such as AN and AP increase under warm–dry environments, while AK is more abundant in cool–humid sites. Stable pools including SOC, TN, TP, and TK segregate along a secondary axis, suggesting distinct controls on nutrient lability versus stability. Stands of Vigor Class II, which frequently occur in warmer and drier locations within the sampled region, exhibit the lowest SMF and highest TVN. This pattern suggests a critical loss of functional resilience during intermediate degradation. Importantly, this elevated TVN may serve not only as an indicator of current instability but also as an early-warning signal of impending functional collapse, consistent with theory linking rising variability to critical transitions.

To clarify the direct drivers underlying these patterns, PLS-PM was applied. Although meteorological variables are strongly correlated with soil nutrient composition in the ordination space, PLS-PM identifies intrinsic soil nutrient status, represented by a composite index of SOC, TN, and TP, as the primary direct correlate of both SMF and TVN. Climate shows no significant direct effect on either response variable once tree vigor and soil conditions are accounted for. These findings suggest that while continued protection of healthy Class I individuals remains essential, priority should be given to targeted ecological rehabilitation of Class II stands to disrupt degradation feedbacks before irreversible thresholds are crossed. This approach is justified by the central role of soil nutrient status in regulating both multifunctionality and stability, thereby highlighting the need to integrate multifunctionality and temporal stability as complementary dimensions of ecosystem health in wild fruit forest conservation, with TVN offering particular value as a leading indicator of resilience loss.

## Data Availability

The original contributions presented in the study are included in the article/**Supplementary Material**. Further inquiries can be directed to the corresponding authors.
