# Supplementary material for: Soil multifunctionality and temporal variability of nutrients across vigor classes in wild apple trees (Malus sieversii)
Source: Front Plant Sci. 2026 Jan 12;16:1711464. doi: 10.3389/fpls.2025.1711464 (PMC12832858; doi:10.3389/fpls.2025.1711464)
Supplement: Supplementary file 1 [file Table1.docx]

# Load necessary packages

library(plspm)

library(dplyr)

library(tidyr)

library(ggplot2)

# Read data

df <- read.csv("date.csv")

# --- CORE MODIFICATION: Create dummy variables for growth vigor ---

df$Vigor_II <- ifelse(df$GrowthVigor == 2, 1, 0)

df$Vigor_III <- ifelse(df$GrowthVigor == 3, 1, 0)

# --- KEY MODIFICATION: Create composite soil fertility index ---

df$Soil_Fertility_Index <- scale(df$SOC) + scale(df$TN) + scale(df$TP)

# --- KEY CORRECTION: Create composite climate stress index (warm-dry = high values) ---

# Note: Subtracting standardized precipitation so low precipitation contributes positive values (i.e., drought stress)

df$Climate_Index <- scale(df$AT) - scale(df$Prep)

# Check descriptive statistics of composite indices

cat("Descriptive statistics for Soil Fertility Index:\n")

print(summary(df$Soil_Fertility_Index))

cat("Standard deviation of Soil Index:", sd(df$Soil_Fertility_Index), "\n\n")

cat("Descriptive statistics for Climate Index:\n")

print(summary(df$Climate_Index))

cat("Standard deviation of Climate Index:", sd(df$Climate_Index), "\n\n")

# Check correlation between AT and Prep

climate_cor <- cor(df[, c("AT", "Prep")], use = "complete.obs")

cat("Correlation between Air Temperature and Precipitation:\n")

print(round(climate_cor, 3))

# --- Update indicator list using climate composite index ---

comdata <- list(

Climate = "Climate_Index",

Soil = "Soil_Fertility_Index",

GrowthVigor_II = "Vigor_II",

GrowthVigor_III = "Vigor_III",

TVN = "TVN",

SMF = "SMF"

)

# --- Latent variable order ---

latent_vars <- c("Climate", "Soil", "GrowthVigor_II", "GrowthVigor_III", "TVN", "SMF")

# Create path matrix

dat_path <- matrix(0,

nrow = length(latent_vars),

ncol = length(latent_vars),

dimnames = list(latent_vars, latent_vars))

# Set causal pathways

dat_path["TVN", "Climate"] <- 1

dat_path["TVN", "Soil"] <- 1

dat_path["TVN", "GrowthVigor_II"] <- 1

dat_path["TVN", "GrowthVigor_III"] <- 1

dat_path["SMF", "Climate"] <- 1

dat_path["SMF", "Soil"] <- 1

dat_path["SMF", "TVN"] <- 1

# Ensure strictly lower triangular matrix (no self-loops, no upper triangular paths)

dat_path <- dat_path * lower.tri(dat_path, diag = FALSE)

# Measurement modes: All reflective (mode A)

dat_modes <- rep("A", length(comdata))

# Run PLS-PM model

set.seed(123)

dat_pls <- plspm(df, dat_path, comdata, modes = dat_modes,

boot.val = TRUE, br = 200)

# View results

summary(dat_pls)

# Plot path diagram of causal relationships

innerplot(dat_pls, colpos = 'red', colneg = 'blue', show.values = TRUE, lcol = 'gray', box.lwd = 0)

# Extract path coefficients

inner_model <- dat_pls$inner_model

# Extract path coefficients, removing Intercept information

path_coefficients <- bind_rows(lapply(names(inner_model), function(name) {

data.frame( Predictor = rownames(inner_model[[name]]),

Response = name,

Estimate = inner_model[[name]][, "Estimate"],

StdError = inner_model[[name]][, "Std. Error"],

tValue = inner_model[[name]][, "t value"],

pValue = inner_model[[name]][, "Pr(>|t|)"] )})) %>%

filter(Predictor != "Intercept") # Remove Intercept information

# Extract R² values

r_squared <- as.data.frame(dat_pls$inner_summary) %>%

select(Type, R2) %>%

filter(Type == "Endogenous") %>%

mutate(Response = rownames(.)) %>%

select(Response, R2)

# Merge R² values with path coefficients table

path_coefficients <- path_coefficients %>%

left_join(r_squared, by = "Response") %>%

arrange(Response, Predictor) %>%

mutate(Significance = ifelse(pValue < 0.001, "***",

ifelse(pValue < 0.01, "**",

ifelse(pValue < 0.05, "*", "ns"))))

# Print formatted path coefficients and R² values table

print(path_coefficients)
